# High-throughput design of bacterial anti-sense RNAs using CAREng

**DOI:** 10.1093/bioadv/vbac069

**Published:** 2022-09-27

**Authors:** Jazmin Romero, Md Tanvir Islam, Ryan Taylor, Cathryn Grayson, Andrew Schoenrock, Alex Wong

**Affiliations:** Research Computing Services, Carleton University, Ottawa K1S 5B6, Canada; Research Computing Services, Carleton University, Ottawa K1S 5B6, Canada; Research Computing Services, Carleton University, Ottawa K1S 5B6, Canada; Department of Biology, Carleton University, Ottawa K1S 5B6, Canada; Research Computing Services, Carleton University, Ottawa K1S 5B6, Canada; Department of Biology, Carleton University, Ottawa K1S 5B6, Canada; Institute for Advancing Health Through Agriculture, Texas A&M University, College Station, TX 77845, USA; Department of Plant Pathology and Microbiology, Texas A&M University, College Station, TX 77843, USA

## Abstract

**Summary:**

Short RNA (sRNA) modulation of gene expression is an increasingly popular tool for bacterial functional genomics. Antisense pairing between an sRNA and a target messenger RNA results in post-transcriptional down-regulation of a specific gene and can thus be used both for investigating individual gene function and for large-scale genetic screens. sRNAs have several advantages over knockout libraries in studies of gene function, including inducibility, the capacity to interrogate essential genes and easy portability to multiple genetic backgrounds. High-throughput, systematic design of antisense RNAs will increase the efficiency and repeatability of sRNA screens. To this end, we present CAREng, the Computer-Automated sRNA Engineer. CAREng designs antisense RNAs for all coding sequences in a given genome, while checking for potential off-targets.

**Availability and implementation:**

CAREng is available as a Python script and through a web portal (https://caren.carleton.ca).

**Supplementary information:**

Supplementary data are available at *Bioinformatics Advances* online.

## 1 Introduction

Laboratory investigations of gene function rely heavily on the ability to perturb specific genes, either by removing them entirely (gene ‘knockouts’) or by reducing their expression (‘knockdowns’). Such approaches are useful both for targeted studies of individual genes and for large-scale screens. Knockouts are the primary tool for genetic screens in model bacteria and yeast—defined knockout libraries are available for multiple species, as are protocols for transposon-based random mutagenesis. In mouse and human cell lines, by contrast, genetic screens are typically accomplished using short-interfering RNAs that reduce gene expression.

Short RNA (sRNA) modulation of gene expression is an increasingly popular tool for bacterial functional genomics (Bhatnagar *et al.*, 2020; Lee *et al.*, 2011; Sharma *et al.*, 2012; Villa *et al.*, 2018; Yoo *et al.*, 2013). Endogenous sRNAs are part of the normal regulatory machinery of the bacterial cell (Jorgensen *et al.*, 2020), with hundreds of known or predicted sRNAs in the model bacterium *Escherichia coli*. sRNAs alter messenger RNA (mRNA) translation by antisense binding to the target mRNA, usually resulting in down-regulation via physical interference with the ribosome, and/or mRNA degradation. For use in functional genomic applications, the antisense region of an endogenous sRNA is modified to bind to a new target mRNA. sRNA screens have been used in a variety of applications, such as the identification of novel antibiotic targets (Bhatnagar *et al.*, 2020; Lee *et al.*, 2011).

Large-scale genetic screens using sRNAs have largely used randomized sRNA libraries (Bhatnagar *et al.*, 2020; Noro *et al.*, 2017; Sharma *et al.*, 2012). While effective, randomization can be inefficient, since many randomized constructs may have no matches in a given transcriptome. Future screening efforts will be made more efficient by the systematic design of sRNAs, with one or more specific sRNAs for every gene in a target genome. In addition to greater efficiency, the systematic design ensures that sRNAs match their targets perfectly. Finally, off-target effects, whereby an sRNA knocks down genes other than its intended target, can be mitigated by searching for other sequence matches in the genome, and through the use of multiple sRNAs for each target gene. To enable the design of systematic sRNA libraries, we report CAREng, the Computer-Automated asRNA Engineer. CAREng designs anti-sense RNA (asRNA) sequences for all (or a subset of) transcripts in an annotated genome while checking for potential off-target binding. These asRNA sequences can then be incorporated into an appropriate sRNA construct. CAREng is available both as a web application and as a stand-alone Python command-line tool.

## 2 Description of CAREng

### 2.1 Scope and workflow

CAREng accepts as input an annotated genome file (EMBL or GenBank format) and an optional list of target genes; if no target list is provided, then asRNAs are designed for all annotated coding sequences in a genome.

**Fig. 1.**
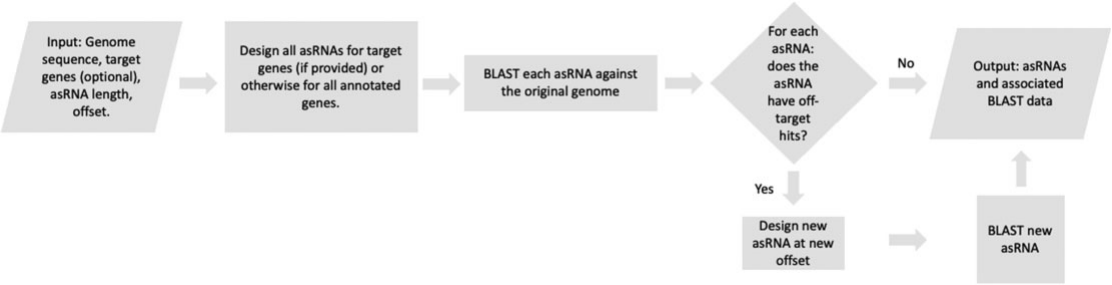
Flow diagram for CAREng’s design of asRNA sequences

The overall workflow is presented in Figure 1. First, asRNAs are designed for every annotated gene, with a user-specified length and distance upstream from the start codon; defaults are 21 nucleotides in length and a -8 nucleotide offset to overlap the typical Shine–Dalgarno (SD) position, reflecting design rules from experimental data (e.g. Hoynes-O’Connor and Moon, 2016). Users wishing to avoid binding to the SD sequence should use an offset of +7 or greater, assuming a 21 nt asRNA. asRNAs are designed as perfect reverse complements for the specified region. Next, we look for off-targets by BLASTing every asRNA against the whole genome (Camacho *et al.*, 2009). BLAST parameters (e-value, percent identity) can be adjusted according to the user’s preferences. Those asRNAs with off-target hits are redesigned at a new offset, and off-targets are again queried by BLAST. Note that we do not carry out additional cycles of asRNA redesign and BLAST: in the case of recent gene duplicates that have high sequence identity, continuing the cycle may result in a long loop and would eventually ‘walk’ the asRNA out of the relevant gene.

**Fig. 2.**
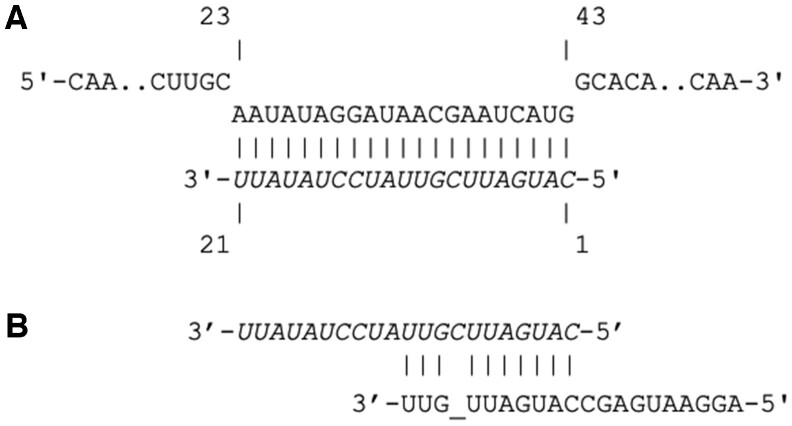
(**A**) Example asRNA against the fliC transcript, designed by CARENg. The designed asRNA is indicated in italics, and the endogenous transcript is in plain font. The RNA–RNA alignment was generated using IntaRNA (Mann *et al.*, 2017). (**B**) Alignment of the CAREng-designed asRNA (italics) with the published fliC-targeting sRNA Spot42-24 (plain) from Sharma *et al.* (2012)

An output file is generated in Microsoft Excel or .csv format, giving the final list of asRNAs, as well as summaries of all BLAST searches. As a use case, we provide the full list of CAREng-designed asRNAs for *E.coli* K-12 (MG1655), a standard laboratory strain of *E.coli*, in Supplementary Table S1. An example asRNA designed by CAREng to target the *fliC* transcript is shown in Figure 2. Notably, this asRNA matches well with a published *fliC*-inhibiting sRNA (Fig. 2B; Sharma *et al.*, 2012).

### 2.2 Implementation and availability

CAREng is implemented in Python 3; we have confirmed compatibility with Python >= 3.6.9. The stand-alone operation of CAREng requires a local installation of BLAST (Camacho *et al.*, 2009) and of BioPython (Cock *et al.*, 2009). A web version of CAREng has been deployed using the Flask framework v1.1.1 (https://flask.palletsprojects.com/) for the backend, Celery v.4.4.7 (https://docs.celeryproject.org) and Redis 3.5.3 (https://redis.io/) for queue management and the React v. 17.0.2 library (https://reactjs.org/) for the front-end. It is available at https://caren.carleton.ca/.

## 3 Discussion

Functional genomic screens in bacteria have typically relied on knockout libraries—strain collections harboring one or more knockout alleles of every non-essential gene in a genome (e.g. Baba *et al.*, 2006; Jacobs *et al.*, 2003). While knockout libraries have provided fundamental insights into gene function and genetic interaction networks, they have several drawbacks: essential genes are by definition not included in knockout libraries, and interrogation of a new genetic background requires the construction of a new library. sRNA screening provides a powerful complement to conventional knockout libraries by addressing both of these shortcomings. Essential genes can be investigated through the use of inducible sRNA constructs (e.g. Lee *et al.*, 2011), and sRNA vectors can be easily introduced into any compatible host strain. Thus, sRNA screening provides a useful complement to knockout-based approaches.

Genome-wide sRNA screens in bacteria have so far relied on randomized libraries (Bhatnagar *et al.*, 2020; Noro *et al.*, 2017; Sharma *et al.*, 2012). Randomized libraries, while relatively easy to implement, are inefficient—many randomized sequences may have no targets in the genome, while others may bind their targets inefficiently and/or bind to multiple target mRNAs. Systematic design of asRNAs alleviates these issues since every designed sequence will have a known, defined target with a high degree of complementarity.

Automated design of antisense sequences is essential for the construction of systematic sRNA libraries in expression vectors suitable for a given host. Nonetheless, no tool is currently available for the genome-wide design of asRNA sequences. CAREng provides this functionality in both stand-alone and web versions, and should thus aid in the development of new functional genomic screens.

## Funding

This work was supported by the Natural Sciences and Engineering Research Council of Canada (RGPIN-2018-05340), CANARIE and Carleton University.

*Conflict of Interest*: none declared.

## Software and data availability

Source code, installation instructions and a web tutorial are available as Supplementary information and at https://caren.carleton.ca/more-information.

## Supplementary Material

vbac069_Supplementary_Data
