# Supplementary material for: High-throughput design of bacterial anti-sense RNAs using CAREng
Source: Bioinform Adv. 2022 Sep 27;2(1):vbac069. doi: 10.1093/bioadv/vbac069 (PMC9710602; doi:10.1093/bioadv/vbac069)
Supplement: vbac069_Supplementary_Data [file vbac069_supplementary_data.zip › Supplement/Installation instructions.docx]

**How to run the program in your local machine**

**Prerequisites**

- Blast: If blast is not already installed, install blast in your local machine by following these instructions:

https://www.ncbi.nlm.nih.gov/books/NBK279671/

- Python:

1. Check if python is installed on your local machine by opening a command window and typing: *python --version*
2. If python is installed, the output should include the version of python that is installed in your machine. For example: *Python 2.7.16*
3. If python is not installed, you can follow these directions to install it. https://www.python.org/downloads/

**Run CARENg locally**

1. Download the latest release of the code.
2. Go to the directory where the CARENg code (srna directory) is located.
3. Create a virtual environment for python by typing: *python3 -m venv env* or by following these instructions: https://docs.python.org/3/library/venv.html
4. Activate your virtual environment by typing: *source ./env/bin/activate*
5. Install the requirements for the environment: *pip install -r requirements.txt*
6. The program to be run is *main.py* and the parameters that it receives are below.

Here is the order in which the program receives the parameters:

*python main.py sequence_file format_sequence shift_position length expected_cutoff identity_percentage_cutoff [-t TAGS] [-r RECOMPUTE]*

- Position Arguments:
  - sequence_file: Sequence file that contains the genome (including absolute path)
  - format_sequence: Format of the sequence file (e.g., genbank or embl)
  - shift_position: Shift position to compute the asRNAs
  - length: asRNAs length
  - expected_cutoff: Expected cutoff when blasting asRNAs against input genome
  - identity_percentage_cutoff: Percentage of identity cutoff used when blasting asRNAs against input genome (a value between 0 and 1)
- Optional arguments:
  - -h, --help: Shows help about how the program usage
  - -t TAGS, --tags TAGS: Excel file that includes the locus/gene tags to compute the asRNAS
  - -r RECOMPUTE, --recompute RECOMPUTE Shift position when recomputing asRNAS with hits

**Important:** The set of tags should follow the format as illustrated in the sample file tags_k12.xlsx. That is, the tags file should contain the following headers and in that order:

Gene_Tag Locus_Tag

**Output:** The program will export the computed asRNAs into an excel file which will be located in the subdirectory *sequences* under the *srna* directory. The name of the output file has this format sequence_file_datetime_asrna.xlsx. In addition, CARENg also exports the set of gene and locus tags of the asRNAs that contain offset-hits in the genome. Notice that this file could be used as input to the program to recompute asRNAs (parameter -T). The name of the tag file has this format sequence_file_datetime_tags.xlsx.

**Examples:**

The directory *sequences* in the directory *srna* contains some sample genome sequences: K12.gb and JWGZ01.1.gbff. It also includes some samples of locus/gene tags for these sequences.

- Example 1: Suppose that you would like to compute **all** asRNAs for the sequence K12.gb. The format for this file is genbank. The offset position to compute the asRNAS would be -10 and the length of the asRNAS would be 19. The expected_cutoff and identity_percentage_cutoff are 0.01 and 0.8, respectively. Additionally, for the asRNAs that contain offset-hits in the genome, the program should recompute these asRNAS with a position of -15. Assume that the program was installed in the following path: /home/srna. Therefore, the sequences are located at directory /home/srna/sequences. For this example, the program should be executed like this:

python main.py /home/srna/sequences/K12.gb genbank -10 19 0.01 0.8 -r -15

The output of the program could look like this: K12.gb_03-08-2021 15:55:32_srna.xlsx and K12.gb_03-08-2021 15:55:34_tags.xlsx.

- Example 2: Suppose that you would like to compute the asRNAs for *a set* of gene/locus tags given in a file. The input file is JWGZ01.1.gbff and the set of tags is in tags_jwz.xlsx. The format for this file is genbank. The position to compute the asRNAs would be -8 and the length of the asRNAs would be 21. The expected_cutoff and identity_percentage_cutoff are 0.01 and 0.8, respectively. Additionally, for the asRNAs that contain hits in the genome, the program should recompute these asRNAs with a position of -10. Assume that the program was installed in the following path: /home/srna. Therefore, the sequences are located at directory /home/srna/sequences. For this example, the program should be executed like this:

python main.py /home/srna/sequences/JWGZ01.1.gbff genbank -8 21 0.01 0.8 -r -10 -t home/srna/sequences/tags_jwz.xlsx

The output of the program could look like this: JWGZ01.1.gbff_03-08-2021 16:03:55_srna.xlsx and JWGZ01.1.gbff_03-08-2021 16:03:55_tags.xlsx

**References**

[1] https://blast.ncbi.nlm.nih.gov/Blast.cgi?CMD=Web&PAGE_TYPE=BlastDocs&DOC_TYPE=FAQ

[2]https://ase.tufts.edu/chemistry/walt/sepa/Activities/BLASTpractice.pdf
