# Supplementary material for: High-throughput design of bacterial anti-sense RNAs using CAREng
Source: Bioinform Adv. 2022 Sep 27;2(1):vbac069. doi: 10.1093/bioadv/vbac069 (PMC9710602; doi:10.1093/bioadv/vbac069)
Supplement: vbac069_Supplementary_Data [file vbac069_supplementary_data.zip › Supplement/Tutorial/Tutorial.docx]

**CARENg – Tutorial**

In this tutorial, we detail the use of the CARENg webserver to design antisense RNAs (asRNAs). We provide two examples:

1. The design of asRNAs for all coding sequences in a genome, and
2. The design of asRNAs for a user specified subset of coding sequences.

For both examples, we will use the *Escherichia coli* K-12 (MG1655) genome. To get started, download the GenBank formatted version of the genome from NCBI. Make sure to select “GenBank (full)” under the format menu (Figure 1). At the time of writing, the link for the most recent version (NC_000913.3) is: <https://www.ncbi.nlm.nih.gov/nuccore/NC_000913.3> Alternatively, the sequence file is included with the supplemental material as “K12.gb”.


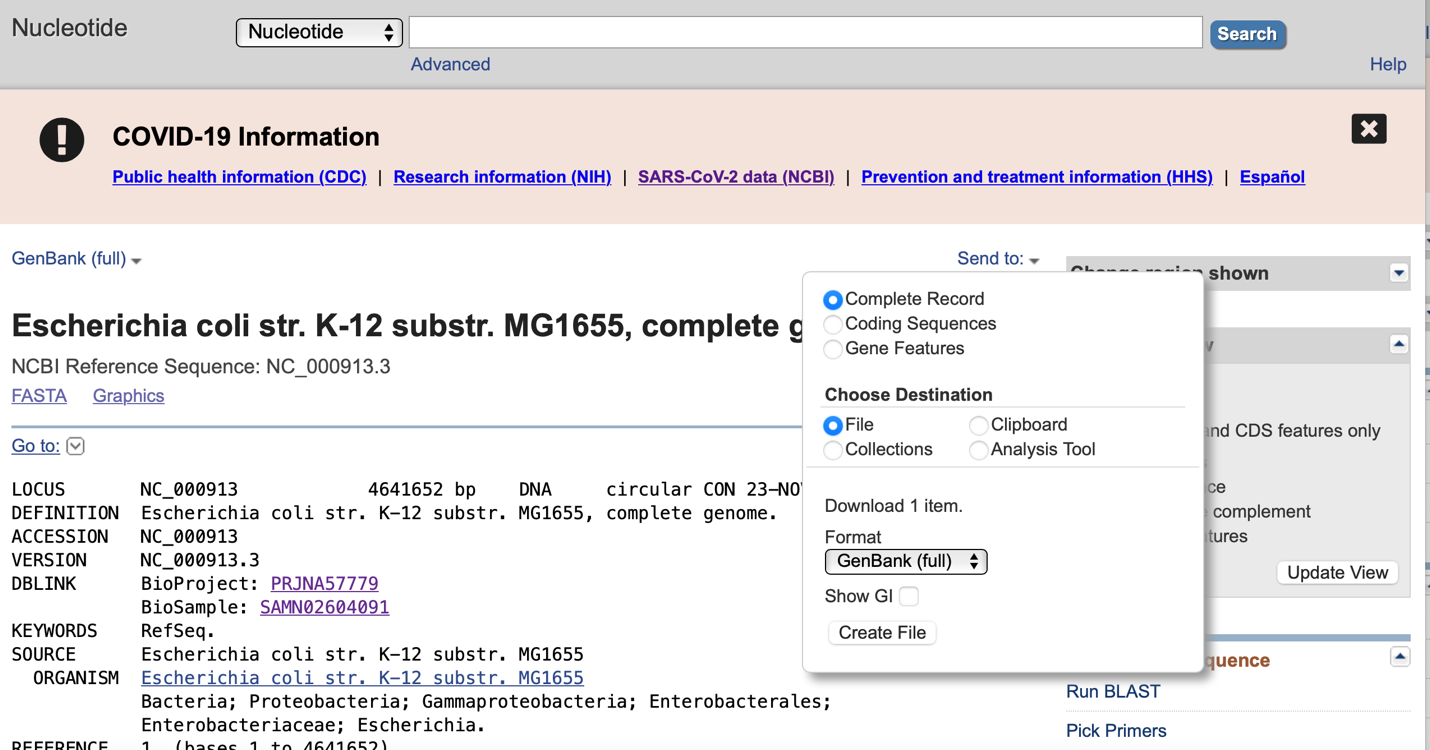


Figure 1. Downloading the GenBank formatted genome from NCBI.

**Example 1. Design of asRNAs for all coding sequences.**

In this example, we will design asRNAs for all coding sequences in the *E. coli* K-12 (MG1655) genome. See above for instructions on how to download the GenBank formatted genome file.

1. Navigate your browser to the CARENG homepage: <https://CAREn.carleton.ca>
2. Click “Choose File”, and upload the genome file.
3. In the “Format” dropdown menu, select “genbank”.
4. For this example, leave all settings at their defaults.
5. Press “Run”.
6. Job progress, as well as recent jobs, will be shown as below.


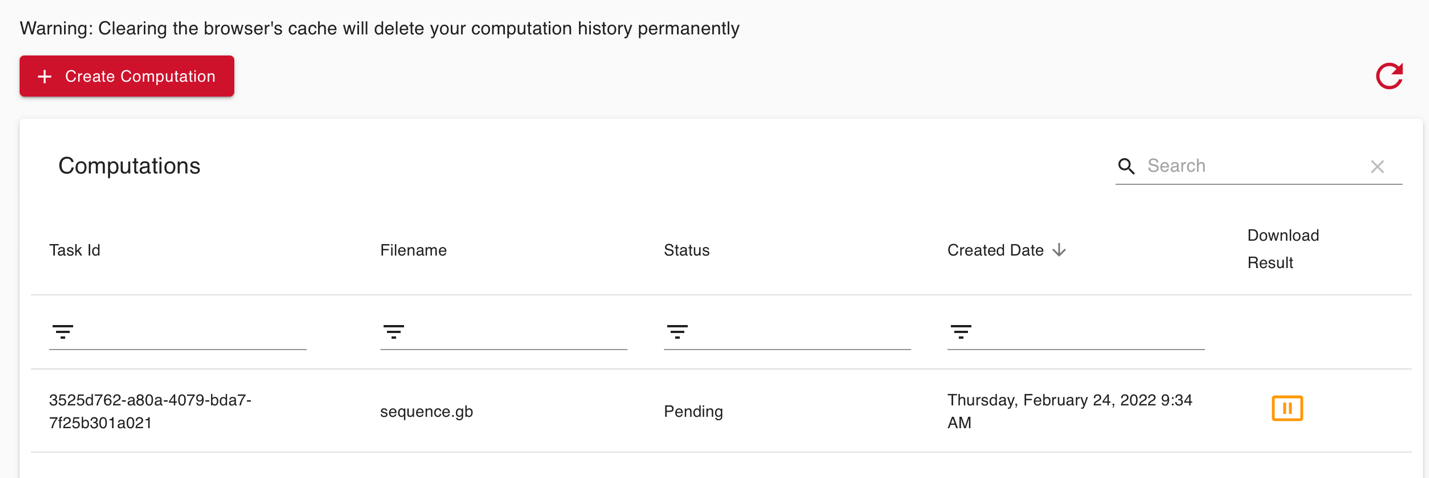


1. Press the “refresh” button (**not** your brower’s refresh button) to update job status.
2. Download the result file.

**Example 2. Design of asRNAs for a subset of coding sequences.**

In this example, we will design asRNAs for a subset of coding sequences in the *E. coli* K-12 (MG1655) genome. See above for instructions on how to download the GenBank formatted genome file.

You will need to prepare a file listing which coding sequences (“tags”) will be used. You can download a template file from the Settings menu on the CARENg homepage:


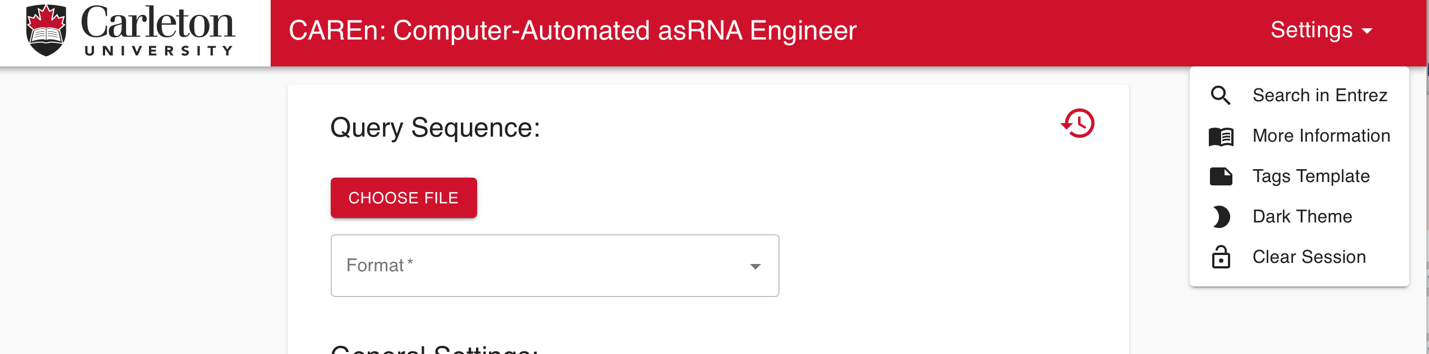


On the template, you need only fill out the Gene_Tag OR the Locus_Tag, not both. For this example, we will design asRNAs for three gene tags: gyrA, priA, and gyrB:


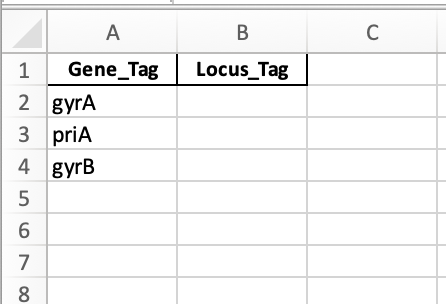


Once you have obtained the source genome, and filled out the template, follow these steps:

1. Navigate your browser to the CARENG homepage: <https://CAREn.carleton.ca>
2. Click “Choose File”, and upload the genome file.
3. In the “Format” dropdown menu, select “genbank”.
4. Click the check box “Compute asRNAs only for these gene tags”.
5. Upload your tag file using the “Choose File” button.
6. For this example, leave all settings at their defaults.
7. Press “Run”.
8. Job progress, as well as recent jobs, will be shown as below.


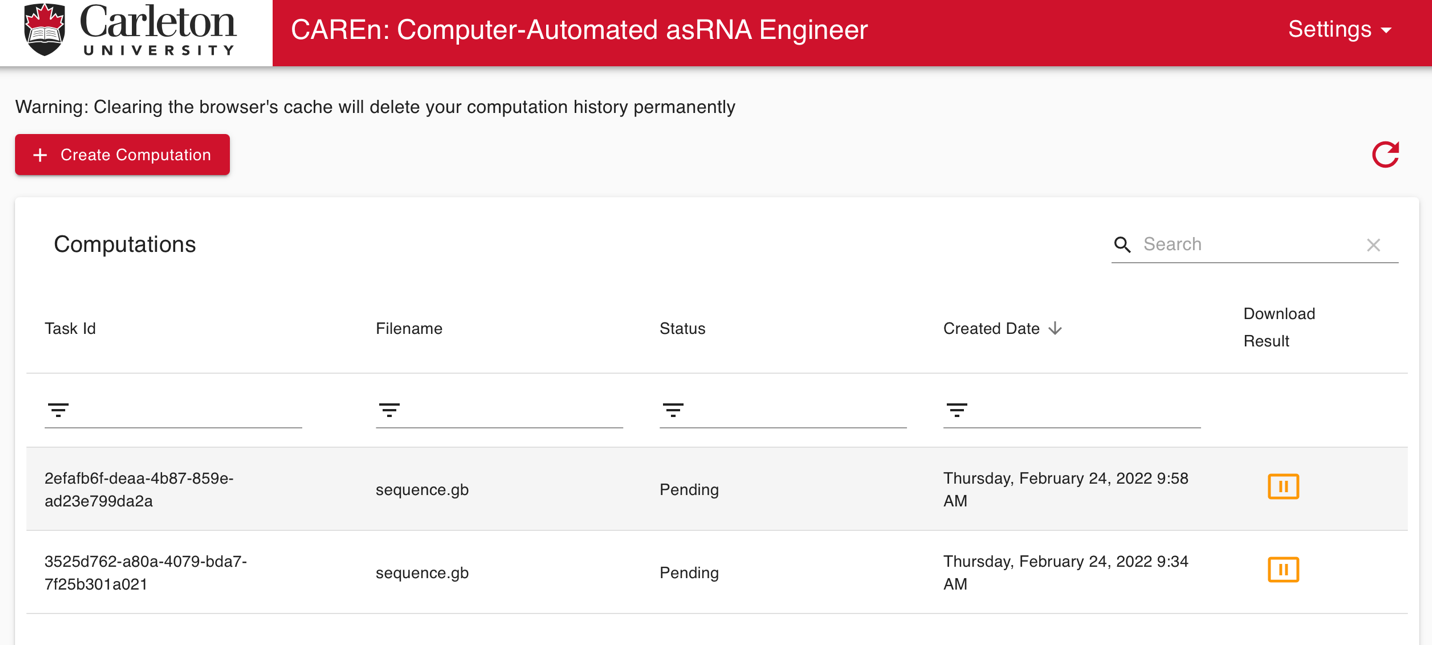


1. Press the “refresh” button (**not** your brower’s refresh button) to update job status.
2. Download the result file.
